# A randomized, controlled study to investigate the efficacy and safety of a topical gentamicin-collagen sponge in combination with systemic antibiotic therapy in diabetic patients with a moderate or severe foot ulcer infection

**DOI:** 10.1186/s12879-018-3253-z

**Published:** 2018-08-02

**Authors:** Ilker Uçkay, Benjamin Kressmann, Sarah Malacarne, Anna Toumanova, Jaafar Jaafar, Daniel Lew, Benjamin A. Lipsky

**Affiliations:** 1 Service of Infectious Diseases, Geneva, Switzerland; 2 Orthopaedic Surgery Service, Geneva, Switzerland; 3 Service of Diabetology and Endocrinology, Geneva University Hospitals and Faculty of Medicine, University of Geneva, Geneva, Switzerland; 4 Division of Medical Sciences, Green Templeton College, University of Oxford, Oxford, UK

**Keywords:** Gentamicin sponge, Diabetic foot infections, Pathogens, Cure, Safety

## Abstract

**Background:**

An adjunctive topical therapy with gentamicin-sponges to systemic antibiotic therapy might improve the healing of infected diabetic foot ulcers (DFUI).

**Methods:**

Single-center, investigator-blinded pilot study, randomizing (1:1) the gentamicin-sponge with systemic antibiotic versus systemic antibiotics alone for patients with DFUI.

**Results:**

We included 88 DFUI episodes with 43 patients in the gentamicin-sponge arm and 45 in the control arm. Overall, 64 (64/88; 73%) witnessed total clinical cure, 13 (15%) significant improvement, and 46 (52%) showed total eradication of all pathogens at the final visit. Regarding final clinical cure, there was no difference in favour of the gentamicin-sponges (26/45 vs. 31/43; *p* = 0.16). However, the gentamicin-sponge arm tended to a more rapid healing. In multivariate analysis adjusting for the case-mix, the variable “gentamicin-sponge” was not significantly associated with “cure and improvement”. Gentamicin-sponges were very well tolerated, without any attributed adverse events.

**Conclusions:**

The gentamicin-sponge was very well tolerated, but did not significantly influence overall cure.

**Trial registration:**

*ClinicalTrials.gov* (NCT01951768). Date 2 April 2013.

**Electronic supplementary material:**

The online version of this article (10.1186/s12879-018-3253-z) contains supplementary material, which is available to authorized users.

## Background

DFUIs are responsible for frequent healthcare visits, a large number of diabetes-related hospital bed-days and a potential predictor of amputation [1]. A recent study [2] of 5.6 billion ambulatory care visits between 2007 and 2013 found that 785 million involved diabetes and 6.7 million were for diabetic foot ulcers (0.3%) or diabetic foot infections (0.5%). Multivariable analyses indicated DFUIs, compared to other diagnoses, were associated with a 6.7 times higher odds of direct emergency referral or hospitalization and 1.5 times more visits in the previous 12 months [2].

Successful treatment of DFUIs requires multidisciplinary management, including attention to wound care, revascularization, off-loading and metabolic abnormalities, as well as antimicrobial therapy and often surgery [3]. These measures are evidence-based or acknowledged by most experts [3]. However, because the infection may still not respond to treatment in a substantial minority of cases, investigators have explored various auxiliary techniques. These may be aimed at enhancing infection cure and/or ulcer healing, and include such methods as patient education and hyperbaric oxygen therapy [3, 4]. Most of these auxiliary measures are not evidence-based sensu *strictu*, but their use is widespread throughout the world [3, 4].

One of these auxiliary approaches is to combine treatment with systemic (intravenous or oral) antibiotic therapy with adjunctive or local antiseptic or antibiotic agents [5]. A recent Cochrane systematic review concluded that randomized controlled trial data on the effectiveness and safety of topical antimicrobial treatments for diabetic foot ulcers is limited by the availability of relatively few, mostly small, and often poorly designed trials [6]. The authors stated “given the high, and increasing, frequency of diabetic foot wounds, we encourage investigators to undertake properly designed randomized controlled trials in this area.”

One product that has been studied for treatment of DFUIs is the combination of a collagen sponge with gentamicin. We previously had performed and published an open-label, multicenter study of 56 patients combining daily application of the gentamicin-collagen-sponge for up to 28 days with systemic antibiotic therapy versus to systemic antibiotic therapy alone in the treatment of moderate DFUI [7]. Although the study failed to meet its primary endpoint (clinical cure at day 7), the gentamicin-sponge showed superior efficacy in terms of eradication of baseline pathogens and, most importantly, a significant higher cure rate at the final visit [7]. Thus, we thought it would be useful to reexamine the potential benefits of the gentamicin-collagen sponge in a similar, but larger single center study, assessing safety and clinical cure rates after more than 1 month instead of 1 week. Our hope was that this pilot study would allow assessing the feasibility of a much larger future (second) multicenter trial.

## Methods

The Geneva University Hospital system is the only public hospital in Geneva and the tertiary center for parts of neighboring France. The institution has been using a clinical pathway [1] for DFUI since 2013, which includes input and consultation from diabetologists, podiatrists, angiologists, infectious diseases physicians, orthopedic and foot surgeons [4], radiologists [8], vascular surgeons, and specialized physiotherapists and surgical nurses (AT) for ortheses and patients’ education.

### Definitions and study criteria

The diagnosis criteria for diabetes mellitus for this study stem from the American Diabetes Association and basically included a glycolysed hemoglobulin A1 level of ≥6.5%, a fasting glycemia level of 7.0 mmol/L, and/or the presence of an anti-diabetic drug as current treatment for diabetes mellitus [9]. We included patients presenting to or patients in our hospital who were found to have a moderate or severe DFUI, based on the criteria developed for IDSA foot infection guidelines [10]. Briefly, a diabetic foot infection is defined as having ≥2 manifestations of inflammation (local swelling or induration, erythema, tenderness or pain, warmth, or purulent discharge (thick, opaque to white or sanguineous secretion). Moderate infection was defined as local DFUI with surrounding erythema > 2 cm, or involving structures deeper than skin and subcutaneous tissues (e.g., abscess, osteomyelitis, septic arthritis, fasciitis) but no evidence of systemic inflammatory response syndrome. Severe infection was defined as local DFUI accompanied by ≥2 systemic signs of inflammatory response syndrome. Cure was defined as absence of microbiological, clinical, and imaging evidence of the original infection. Improvement was defined as a significantly better wound score and a decrease in the number of manifestations of inflammation, however without complete resolution. Regarding the evolution of the wound, we used a custom-made DFUI assessment to describe the evolution of the wound, summing up various elements of a wound in a single wound score (Additional file 1). The elements used for that score were all quantified, and consisted of the wound size, wound depth, the degree of undermining of wound edges, the presence of discharge and pus, erythema, induration, tenderness, pain and local warmth (Additional file 1).

**Fig. 1:**
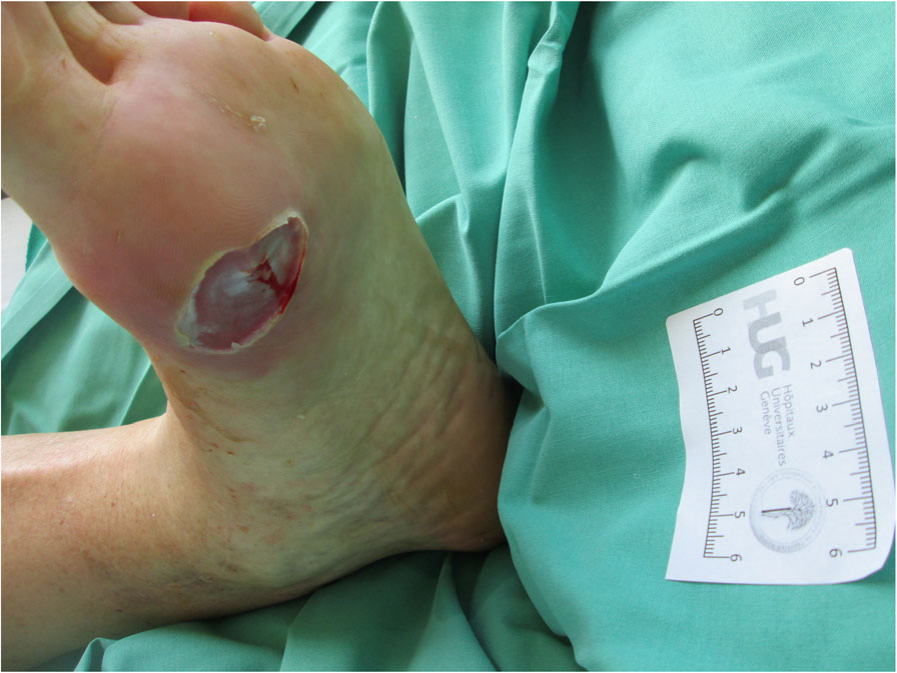
Local infection and ulcer not requiring surgery. Included in study. Photo permitted by patient

**Fig. 2:**
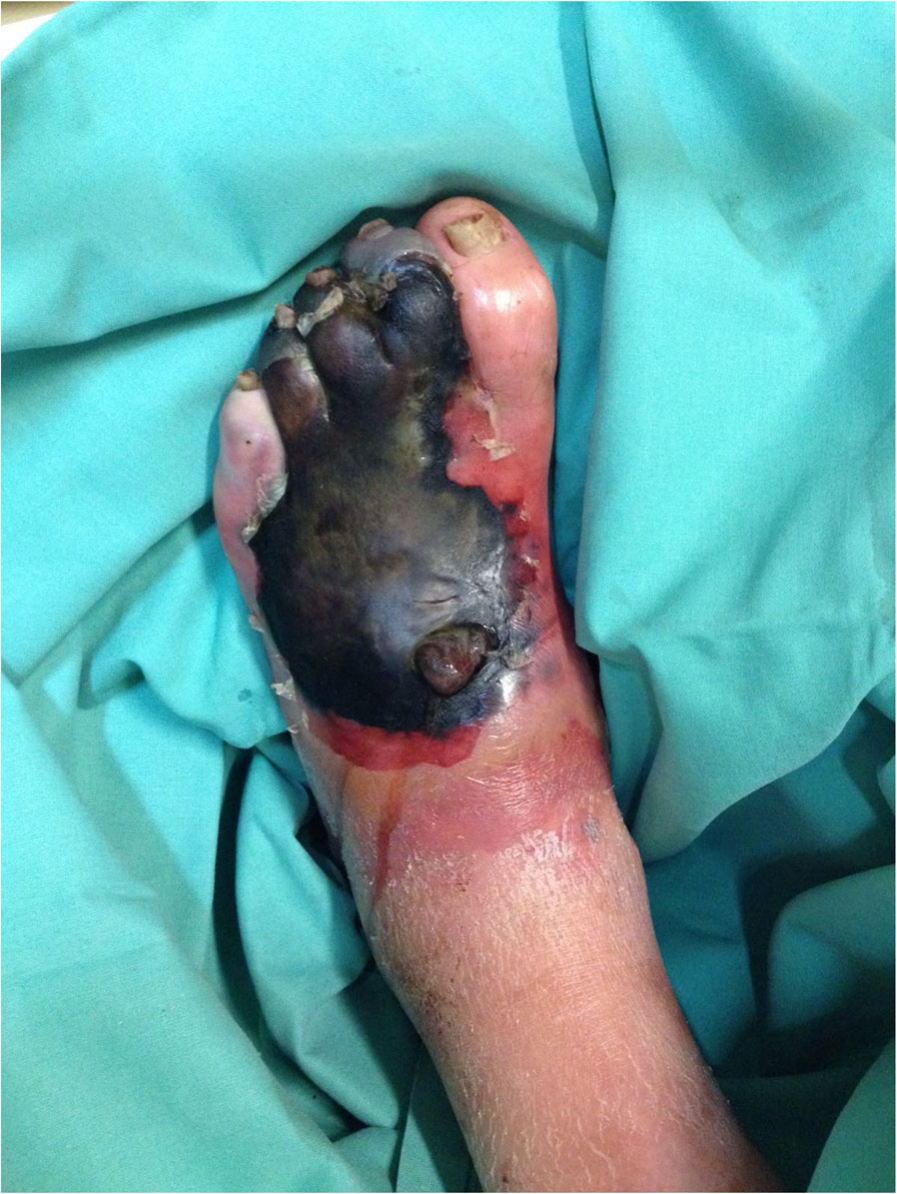
Diabetic foot necrosis with underlying abscess requiring amputation. Excluded from study. Photo permitted by patient

A patient was eligible for study participation if he/she met the following criteria: is aged ≥18; has been diagnosed with diabetes mellitus; has an open foot wound of ≥1 cm^2^ located on or below the malleolus that has findings of infection; has undergone any appropriate surgical intervention needed to remove necrotic and to drain an abscess (Fig. 1); and, if female, is nonpregnant and nonlactating. Of note, surgical debridement was not a must. Patients could participate in the study without prior surgical debridement if there was no clinical need to remove necrosis or to drain an abscess. The exclusion criteria were: a known history of hypersensitivity to gentamicin or bovine collagen [11]; a DFUI associated with prosthetic material or any implanted device; peripheral arterial insufficiency clinically requiring urgent limb revascularization after inclusion; and, having received > 48 h of potentially effective antibiotic therapy and showing improvement in wound infection. Of note, a chronic arterial insufficiency with a clinically preserved foot in absence of vascular necroses was not an exclusion criterion. If a patient had received an antibiotic within 72 h, but deep-tissue culture results indicated that the infecting pathogen was not susceptible to that antibiotic, the patient could be enrolled. Further exclusion criteria were: mild DFUI (as defined by the IDSA criteria [10]); severe immune-suppression; extensive necrosis requiring amputation (Fig. 2); residual osteomyelitis (after any debridement) requiring more than 28 days of antibiotic treatment [1]; a history of myasthenia gravis or other neurological conditions precluding gentamicin use; a history of epilepsy; and, active or recent alcohol or substance abuse.

### Gentamicin-collagen sponges

**Fig. 3:**
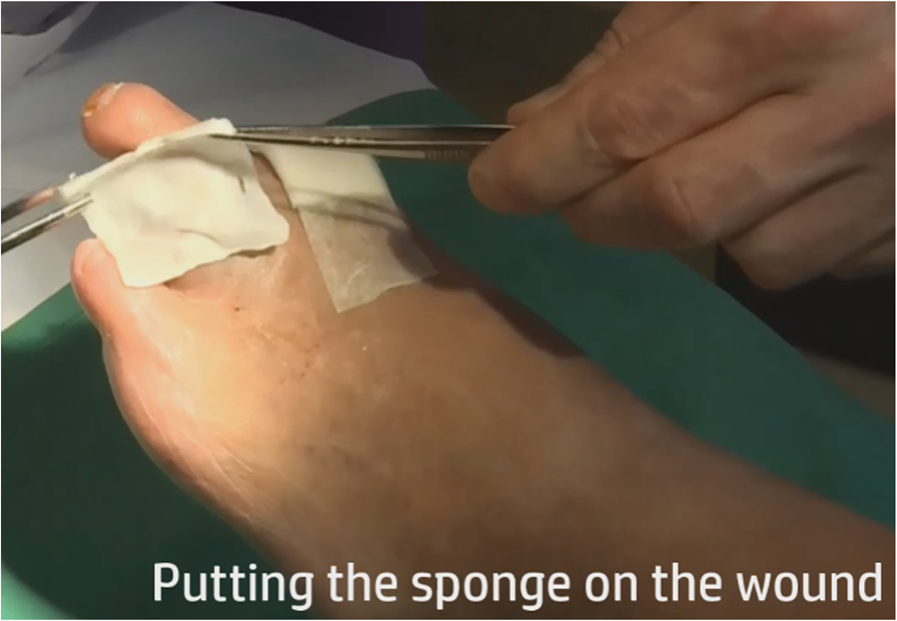
Putting the sponge on the wound. Educational photo with the permission of the patient

Innocoll Pharmaceuticals Ltd. provided us with the bioresorbable gentamicin-collagen sponges. These are 5 × 5 cm in size and contain type 1 bovine collagen and 50 mg of gentamicin sulfate (equivalent to 32.5 mg of gentamicin base), and are commercially available in Switzerland under the trade name GARAMYCIN® Sponge (Fig. 3; unpacked sponge). We made an educational videotape to demonstrate the correct application of the sponge (Additional file 2: Video S1).

### Study conduct

This was a prospective, randomized, investigator-blinded, controlled, single-centre study. Using a blinded allocation scheme, patients were randomized 1:1 to receive systemic antibiotic therapy and standard ulcer care, with either (A) daily application of a gentamicin-sponge, or (B) no-sponge. The clinical investigator (IU or BK) selected the highest severity ulcer (if there was more than one) and determined the number of sponges (up to 4) that a patient required to fully cover the wound. If needed, the patient underwent surgical debridement (or partial amputation) as long as there was residual infected wound. The patient could also undergo limb revascularization if the procedure was performed before they started in the study. We did not impose any study-related conditions regarding the surgical or angiologic techniques provided to the patient.

We allowed enrolled patients to have been on an effective systemic antibiotic regimen for up to 48 h prior to study inclusion. Once the patient was enrolled in the study, the clinical investigator (IU, an infectious diseases physician) prescribed a systemic antibiotic regimen (empiric if there were no culture and sensitivity results, specific if there were), based on a scheme developed specifically for this protocol. For both study arms, the preferred systemic (oral or intravenous) antibiotic treatment was levofloxacin (with or without additional clindamycin if necessary), or amoxicillin-clavulanate. In cases of severe infection or sepsis, we treated the patient with piperacillin-tazobactam or aztreonam, if needed [10]. If infection with an obligate anaerobe was clinically suspected or confirmed [12], we added metronidazole (if the patient was not already receiving clindamycin). If cases with suspected or proven infection with methicillin-resistant staphylococci [13], we added linezolid to the regimen. Standard wound care included sharp debridement (at enrollment and during hospitalization or at clinic visits, if clinically indicated), dressing changes (moisture of 0.9% saline for those not treated with the gentamicin-collagen sponge), pressure off-loading and efforts to optimize glycemic control. Enrolled patients could not be treated with topical antiseptics or antibiotics (other than the gentamicin-sponge), hyperbaric oxygen therapy or vacuum-assisted negative-pressure devices.

Patients were scheduled to receive approximately 14 to 28 days of study treatment. They were seen for follow-up in the hospital (if an inpatient) or if treated as outpatients, in the clinic, weekly (on nominal days 8, 15, 22, and 29) for safety and efficacy assessments. The end-of-treatment visit was defined as the patient’s last visit on which study treatment was formally discontinued for any reason. The final assessment was performed at the test-of-cure visit, approximately 10 days after treatment was discontinued. For each enrolled patient we assessed variables pertaining to demographic characteristics, immune-suppression status, wound microbiology results, surgical procedures, and antibiotic treatment. A microbiological culture of tissue collected from the target ulcer (obtained by debridement, curettage, or biopsy, but not swab) was performed at baseline and at the final visit (if there was ulcers left for debridement). We noted eradication or persistence of baseline pathogens, based on culture results reported by the local laboratory. We incubated all specimens for up to 5 days and processed any isolates according to CLSI recommendations [14], before switching to EUCAST criteria (European Committee) in 2014 [15]. Finally, we made safety assessments at each visit during the study. Investigators specifically inquired about any vestibular disturbances and ototoxicity, such as nausea, tinnitus, vertigo, and decrease in hearing.

A research nurse (BK) and an infectious diseases physician (IU), who both specialized in DFUI, supervised enrollment and follow-up of all patients, distinguished between infection and colonization and between causative pathogens and less virulent microorganisms. A second research nurse (LH) monitored the study. Innocoll Ltd. made a donation to our medical center to support the costs of conducting this pilot study. This donation did not come with any conditions or requirements regarding the academic freedom and scientific publications. We registered the study on *ClinicalTrials.gov* (NCT01951768) on 2 April 2013.

### Statistical analyses

Our primary objective was to determine the effect of the topical gentamicin-collagen sponge in combination with systemic antibiotic therapy compared to systemic antibiotic therapy alone on clinical outcome in the treatment of a moderate or severe DFUI. Secondary objectives were: to determine the effect of the gentamicin-sponge on the eradication of baseline ulcer pathogens; to assess the safety and tolerability of the gentamicin-sponge; and, to determine the rapidity of wound healing over time. Assuming a clinical “clinical cure” in 90% of patients treated with the gentamicin-sponge versus 70% in the control group (no-sponge), we aimed for a sample size of 144 subjects to all 80% power to detect superiority of the gentamicin-sponge arm at the 0.05 significance level.

We performed group comparisons using the Pearson-χ^2^-test, the Fisher-exact, or the Wilcoxon-ranksum-test, as appropriate. We performed an unmatched logistic regression analysis with the outcome “cure”, adjusted for the case mix. We introduced independent variables with a *p* value ≤0.05 in univariate analysis stepwise into the multivariate analysis, except for variables for gentamicin sponges, which we automatically included in the model. We included 12 predictor variables per outcome [16] and checked variables for collinearity and interaction. We used STATA software (9.0, STATA™, USA). *P* values ≤0.05 (two-tailed) were significant.

## Results

### Patients

**Fig. 4:**
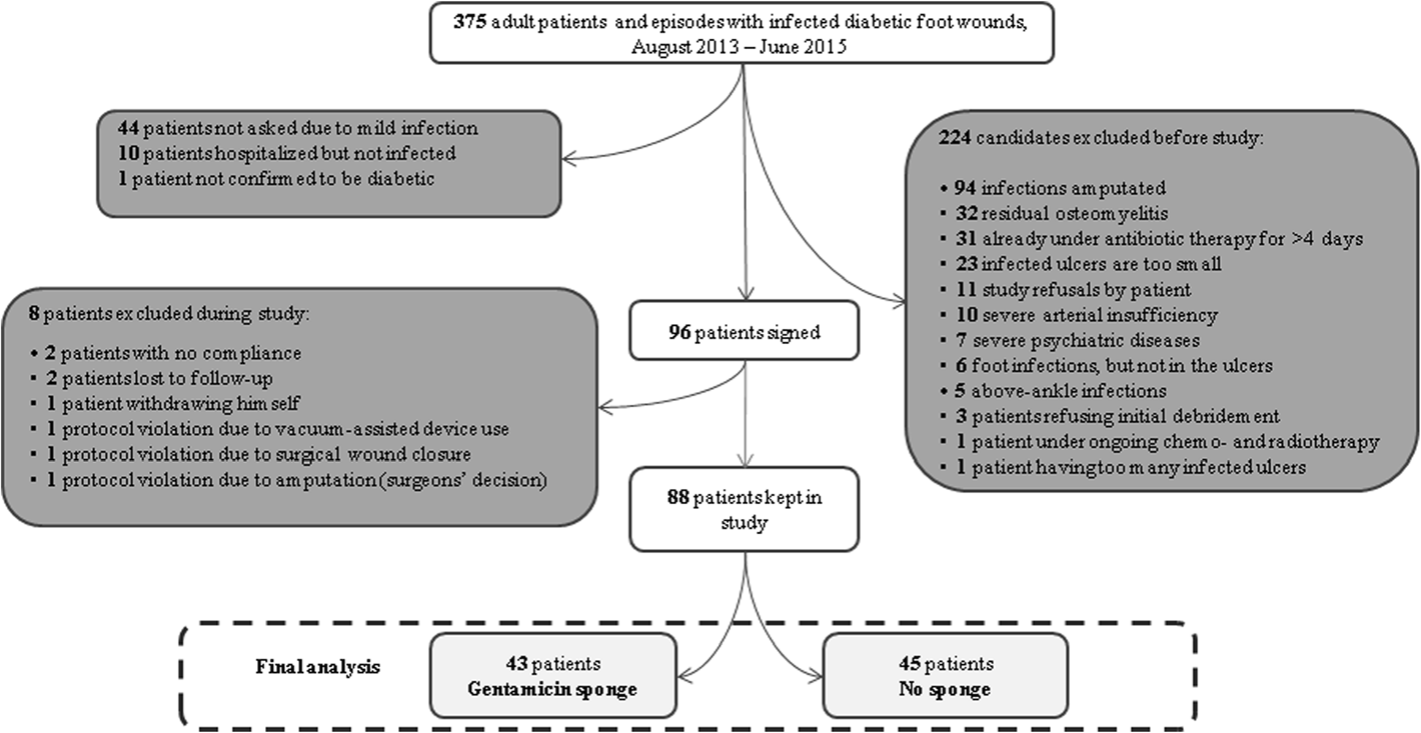
Flowchart of the study inclusion

Over nearly three years (August 2013–June 2015) we screened 375 DFUI cases for inclusion in this study. Of these, we excluded 287 cases because they met one or more of our exclusion criteria (see Fig. 4). The most common reasons for exclusion were that the patient: underwent amputation of the affected area (94 cases); had residual osteomyelitis underlying the DFUI (32); had been treated with antibiotic therapy for > 2 days at the time of screening (31); or had a DFUI that was small than our minimal size (23). Finally, we included 88 (23%) of the screened DFUI cases, 43 of whom were randomized to the gentamicin-sponge arm and 45 to the control arm. Among the included patients, the mean age was 71 and 64% were men. The DFUI was classified as moderate in 77 cases and severe in 11 cases^9^. The location of the DFUI was in the hindfoot in nine DFUI (10%), the midfoot in 27 (31%), and the toes in 52 (59%). In 66 episodes, there was 1 DFUI per foot, in 16 cases two, in 4 cases three, and in 2 cases, four DFUIs.

#### Pathogens, prior surgery and systemic antibiotic therapy

The four most frequently identified pathogens from cultures of the DFUIs were *Staphylococcus aureus* (in 41 episodes, of which eight were methicillin-resistant), streptococci (11), *Escherichia coli* (9), and *Pseudomonas aeruginosa* (5). Polymicrobial infection was noted in 37 episodes (42%), 32 (36%) were predominantly Gram-negative pathogens and in four episodes (5%), there was mixed growth of obligate anaerobic pathogens in the intraoperative samplings. Among all patients the DFUI was treated with systemic antibiotics for a median duration of 21 days (range, 7–42 days); in 49 cases the patient was treated with oral antibiotic therapy from the start, while the others had initial intravenous therapy. The most frequently used antibiotics were co-amoxiclav (*n* = 58), levofloxacin (*n* = 48), linezolid (*n* = 6), metronidazole (n = 4), piperacillin-tazobactam (*n* = 3), and clindamycin (n = 3). The median number of surgical procedures before inclusion was 1; in 20 cases there was a partial foot amputation and 4 patients underwent angioplasty prior to inclusion. Baseline characteristics were similar in the two study arms (see Table 1).

**Table 1:** Comparing patients with infected diabetic foot ulcers; with and without gentamicin-sponges

*n* = 88	No sponges (control arm)		Sponges (investigator arm)
	*n* = 45	p value*	*n* = 43
Female sex	16 (36%)	0.21	10 (23%)
Median age	71 years	0.40	72 years
Median body mass index	28.3 kg/m^2^	0.39	30.3 kg/m^2^
Moderate diabetic foot infection	38 (84%)	0.38	39 (91%)
- Infected ulcer at toe level	23 (51%)	0.12	29 (67%)
Median Wound Score at inclusion	18 points	0.78	18 points
- Median number of infected ulcers	1	0.44	1
Median leukocyte count at inclusion	10.3 G/L	0.24	9.3 G/L
Median serum creatinin level at inclusion	87 umol/L	0.29	106 umol/L
*Staphylococcus aureus (methicillin-susceptible)*	25 (56%)	0.09	16 (37%)
Streptococci	7 (16%)	0.16	12 (28%)
Gram-negative pathogens	14 (31%)	0.30	18 (42%)
- *Pseudomonas aeruginosa*	1 (2%)	0.15	4 (9%)
Median duration of systemic antibiotics	21 days	0.19	21 days
- Oral antibiotic therapy from the start	26 (58%)	0.69	23 (53%)
Median number of prior surgeries	0	0.97	0
- Prior partial amputation	9 (20%)	0.53	11 (26%)
Prior revascularisation^a^	7 (16%)	0.89	7 (16%)
Compliance with off-loading	41 (91%)	0.45	37 (86%)
Total cure & significant improvement	39 (87%)	0.81	38 (88%)
- Total cure; without just improvement	26 (79%)	0.16	31 (94%)
Total pathogen eradication	20 (44%)	0.13	26 (60%)

*Significant *p* values ≤0.05 (two-tailed) are displayed

^a^Revascularisation of any arteria in patient’s history, independently of the present diabetic foot ulcer infection

#### Outcomes

Among the 88 DFUI episodes*,* 64 (64/88; 73%) were classified as clinical cure, 13 (15%) as improvement. Seven cases (8%) were classified as stagnation and 4 (5%) as worsening - each of which was clinically attributed to worsening ischemia during the weeks the patient was in the study (Table 1). There was no significant difference in the combined clinical cure and improvement between those treated with or without the gentamicin-collagen sponge (31/43 [88%] and 39/45 [87%], respectively). This was similar considering cure alone by omitting the outcome “improvement” (31/43 [72%] and 26/45 [58%]) (Pearson-χ^2^-test: *p* = 0.16). Microbiological assessment demonstrated that in 46 (52%) episodes there was total eradication of all pathogens at the test-of-cure visit. In the rest of the episodes, there was either nothing left for tissue sampling or there were new or previously known pathogens detected. There was no significant difference in the rate of pathogen eradication in patients treated with or without the gentamicin-collagen sponges (20/45 and 26/43, respectively; *p* = 0.13).

**Fig. 5:**
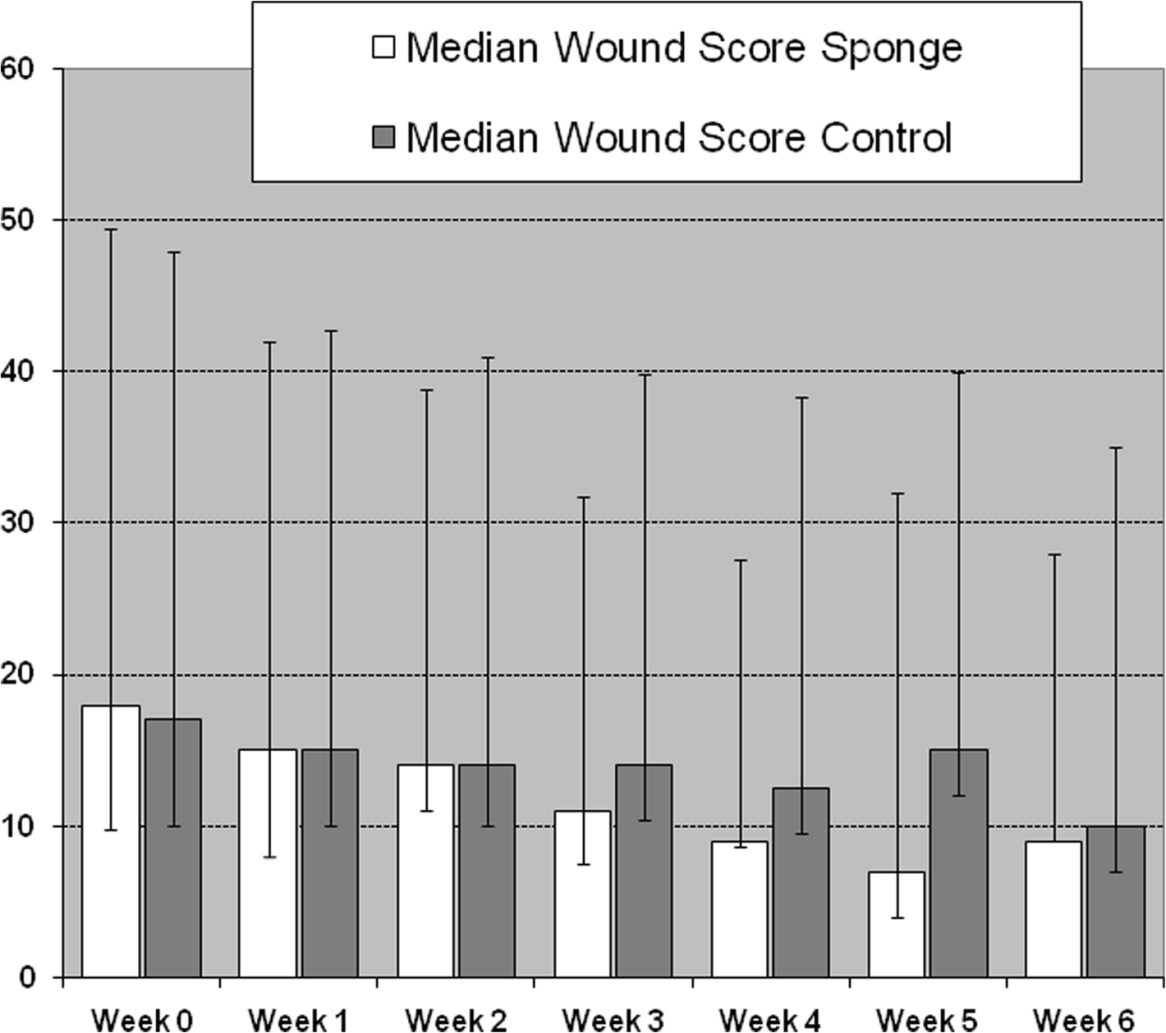
Median wound scores over 6 weeks stratified by study arms

At the final visit, all patients were afebrile and the median leukocyte count decreased from 9.9 G/l on admission to 7.8 G/l. The wound score (Additional file 1) decreased from a median of 18 points at enrollment to a median of 8 points at the final visit. The decrease in the wound score tended to be more rapid in patients treated with the gentamicin-collagen sponge during the study weeks 3 to 5 (Fig. 5). We noted that in 10 cases (11%) pressure off-loading was suboptimal due to the patients’ low (but not non-existent) adherence to using the prescribed device, but these patients remained in the study.

Both the saline-moistened dressings and the gentamicin-collagen sponges were well tolerated, without any attributable adverse events. There were no cases of oto- or neurotoxicity (beyond the known peripheral neuropathy due to diabetes). In the gentamicin-collagen sponge arm, the median serum creatinine levels at inclusion were 109 mmol/L, and 97 mmol/L at the end. Overall, we noted 20 minor adverse events, the most important of which were: gout crisis (1), nosocomial pneumonia (1), and worsening arterial insufficiency (5).

#### Multivariate adjustments

We performed an unmatched logistic regression analysis with the combined outcome parameter “cure & improvement” to adjust for the considerable case-mix (see Table 2). The variable gentamicin-collagen sponge was not significantly associated with “cure & improvement”. The results of the goodness-of-fit-test were not statistically significant and the receiver-operating-curve value was 0.91, highlighting a more than acceptable accuracy of the final model.

**Table 2:** Associations with cure (combined variable of total cure & improvement; unmatched logistic regression)

**n = 88**	Univariate analysis	Multivariate analysis
	Odds ratio (95% confidence interval)	Odds ratio (95% confidence interval)
Body mass index (continuous variable)	0.8 (0.7–1.1)	0.6 (0.3–1.2)
Age (continuous variable)	1.0 (0.8–1.1)	0.8 (0.6–1.2)
Glycated hemoglobulin (continuous variable)	0.8 (0.5–1.4)	n.d.
Duration of antibiotic treatment (continuous variable)	0.9 (0.7–1.2)	1.1 (0.6–1.9)
Gentamicin-sponge use	1.0 (0.1–16.1)	1.0 (0.1–15.8)

Statistically significant results are displayed

*n.d.* not done

## Discussion

Our randomized, controlled trial was designed to determine if, for patients with a DFUI, the addition of a topical gentamicin-collagen sponge to systemic antibiotic therapy plus local wound care and surgery as needed, was beneficial. Our results showed no significant differences, between the group that received the sponge and the group that did not, in the rate of clinical cure, clinical improvement of DFUI or pathogen eradication. Thus, the gentamicin-collagen sponge arm did not prove to be superior to the control arm (no gentamicin-collagen sponge). In our previous study, the gentamicin-collagen sponge group had a higher proportion with total cure and a higher rate of eradication of baseline pathogens [7].

Interestingly, in our patients there was an apparent tendency to a more rapid or more pronounced ulcer healing in the gentamicin-collagen sponge group during the second half of the study (weeks three to five), compared to the control arm. Even if this difference was not statistically significant, a similar finding was noted in two previous studies of the gentamicin-collagen sponge in patients with diabetic foot wounds [7, 17]. Both randomized trials observed a shortened wound healing time in favor of the gentamicin-collagen sponge. In the study by Varga et al. with 50 patients who had undergone a minor foot amputation, the median time difference was 2 weeks [17]. As reported in previous studies [7, 17], the gentamicin-collagen sponge was well tolerated with no adverse effects of concern. Although our results did not show a significant benefit for the gentamicin-collagen sponge as adjunctive therapy for treating DFUIs, we have demonstrated the feasibility of a potential larger multicenter trial.

We terminated this pilot study earlier than scheduled (after enrolling 88 patients instead of the planned 144), because it was more difficult than we expected to find eligible cases based on our enrollment criteria. Among all DFUI patients we screened, despite the fact that only 3% (11/375) declined to participate, only 23% were eligible for enrollment due to exclusion criteria. Using our power calculation that was based on a success of 90% in the sponge arm and a power of 80%, our final sample size would have detected a 17% difference in a sponge superiority study, and 9% difference in a control non-inferiority study (with the difference set at 10%).

There have been many published reports of topical antimicrobial treatments for infected ulcers, including of the diabetic foot. Beyond anecdotal reports [18], available studies have not found any significant benefit of local antiseptic or antibiotic agents in healing the ulcers per se or in preventing infection [19]. These topical agents might have a role in curing superficial infections [7], but they do not appear to add benefit to a concomitant systemic antibiotic therapy in terms of overall cure [20, 21]. A recent Cochrane systematic review and meta-analysis of topical antimicrobial therapy for diabetic foot ulcers concluded that randomized controlled trial data on the effectiveness and safety of topical antimicrobial treatments for diabetic foot ulcers was limited to a relatively few, mostly small, and often poorly designed trials [6]. Indeed, the authors noted that in view of “the high, and increasing, frequency of diabetic foot wounds, we encourage investigators to undertake properly designed randomized controlled trials … to evaluate the effects of topical antimicrobial treatments for … the treatment of infection in these wounds and ultimately the effects on wound healing.”

The antimicrobial effect of the gentamicin-collagen sponge relies on gentamicin, which is delivered by the topical, rather than the more usual systemic, route. Gentamicin and other aminoglycosides can be released from bovine collagen like with the sponge used in our study [11], they can be delivered by the polymer chitosan [22, 23], or by other means such as calcium sulfate pellets [24]. Theoretically, advantages in wound healing times could be associated with the collagen composition of the dressing instead of the gentamicin compound, for which we found no evidence in the literature. Since both compounds are usually mixed together in one sponge, formally the different author groups cannot sharply distinguish between the individual benefits of the compounds. However and logically, the anti-microbiologically active compound is the gentamicin and not the bovine collagen.

Topical anti-infective agents have many potential advantages [5] and can employ innovative agents and approaches to therapy. For example, a novel investigational agent, 1% pexiganan acetate cream, has been studied in mild DFUIs. Pexiganan is a peptide disrupting the integrity of bacterial cell membranes. Two prospective, blinded, randomized, controlled, multicentre trials compared topical pexiganan with oral ofloxacin in a total of 835 (for the two studies combined) DFUIs. The clinical response rate at end of 7–42 days of therapy was 85–90% for both groups; the microbiological response and wound healing rates were equivalent [25]. More recently, however, preliminary results from two large, multicenter, randomized controlled trials comparing topical pexiganan gel to a placebo (the gel vehicle) for mild DFUIs found no significant difference in clinical or microbiological outcomes [26]. Other studies of topical therapy for DFUI have employed a variety of antibiotics (e.g., mupirocin, bacitracin, neomycin, chloramphenicol, polymyxin B, and gentamicin) as well as antiseptics [3].

## Conclusion

In this second pilot study performed by our group, the gentamicin-sponge did not significantly influence overall clinical cure or the eradication of wound pathogens. The gentamicin-sponge was very well tolerated without attributable adverse events. If necessary, our pilot study confirms the feasibility of a much larger second multicenter clinical trial.

## Additional files

Additional file 1: DFUI WOUND SCORE-TARGET INFECTION ( ULCER WITH HIGHEST DFI WOUND (DOC 79 kb)

Additional file 2: Video S1. (MP4 234114 kb)

## Abbreviations

CLSI: Clinical and Laboratory Standard’s Institute
DFUI: Diabetic foot ulcer infections
IDSA: Infectious Diseases Society of America
