# Supplementary material for: A randomized, controlled study to investigate the efficacy and safety of a topical gentamicin-collagen sponge in combination with systemic antibiotic therapy in diabetic patients with a moderate or severe foot ulcer infection
Source: BMC Infect Dis. 2018 Aug 2;18:361. doi: 10.1186/s12879-018-3253-z (PMC6090847; doi:10.1186/s12879-018-3253-z)
Supplement: Supplementary file 1 — DFUI WOUND SCORE-TARGET INFECTION ( ULCER WITH HIGHEST DFI WOUND (DOC 79 kb) [file 12879_2018_3253_MOESM1_ESM.doc]

***DFUI Wound Score-TARGET infection***  *( ulcer with highest DFI wound score)*

**GENERAL PARAMETERS** *(check only one per category)*

**Purulent Drainage Non-purulent Drainage Erythema Induration**

^0^ Absent  ^0^ Absent  ^0^ None  ^0^ None

^3^ Present  ^1^ Mild  ^1^ Mild  ^1^ Mild

^2^ Moderate  ^2^ Moderate  ^2^ Moderate

^3^ Severe  ^3^ Severe/Extreme  ^3^ Severe

**Tenderness (sign) Pain (symptom) Local warmth increase**

^0^  None  ^0^ None  ^0^ Absent

^1^ Mild  ^1^ Mild  ^1^ Mild

^2^ Moderate  ^2^ Moderate  ^2^ Moderate

^3^ Severe/Extreme  ^3^ Severe/Extreme  ^3^ Severe/Extreme **SUBTOTAL**

**PARAMETERS**

**GENERAL MEASUREMENTS** *(check only one per category)*

**Size (cm2) Depth (mm) Undermining (mm)**

^0^ < 1  ^0^ < 5  ^3^ < 2

**^1^** ≥ 1 to 2  *^3^* 5-9  ^5^ 2 to 5

^3^ > 2 to 5  ^7^ 10-20  ^8^ >5

^6^ >5-10  ^10^ >20

^8^ >10 to 30

^10^ > 30 **SUBTOTAL**

**MEASUREMENTS**

**TOTAL WOUND SCORE**

#### 
